# Machine learning-based reproducible prediction of type 2 diabetes subtypes

**DOI:** 10.1007/s00125-024-06248-8

**Published:** 2024-08-21

**Authors:** Hayato Tanabe, Masahiro Sato, Akimitsu Miyake, Yoshinori Shimajiri, Takafumi Ojima, Akira Narita, Haruka Saito, Kenichi Tanaka, Hiroaki Masuzaki, Junichiro J. Kazama, Hideki Katagiri, Gen Tamiya, Eiryo Kawakami, Michio Shimabukuro

**Affiliations:** 1 Department of Diabetes, Endocrinology, and Metabolism, Fukushima Medical University School of Medicine, Fukushima, Japan; 2 Department of Diabetes, Metabolism and Endocrinology, Tohoku University Graduate School of Medicine, Miyagi, Japan; 3 Department of AI and Innovative Medicine, Tohoku University School of Medicine, Miyagi, Japan; 4 Shimajiri Kinsermae Diabetes Care Clinic, Okinawa, Japan; 5 Department of Statistical Genetics, Osaka University Graduate School of Medicine, Osaka, Japan; 6 Tohoku Medical Megabank Organization, Tohoku University, Miyagi, Japan; 7 Department of Nephrology and Hypertension, Fukushima Medical University School of Medicine, Fukushima, Japan; 8 Division of Endocrinology and Metabolism, Second Department of Internal Medicine, University of the Ryukyus Graduate School of Medicine, Okinawa, Japan; 9 Department of Artificial Intelligence Medicine, Graduate School of Medicine, Chiba University, Chiba, Japan; 10 Advanced Data Science Project, RIKEN Information R&D and Strategy Headquarters, RIKEN, Yokohama, Japan

**Keywords:** Clustering, Diabetes subtypes, Machine learning, Random forest, Type 2 diabetes

## Abstract

**Aims/hypothesis:**

Clustering-based subclassification of type 2 diabetes, which reflects pathophysiology and genetic predisposition, is a promising approach for providing personalised and effective therapeutic strategies. Ahlqvist’s classification is currently the most vigorously validated method because of its superior ability to predict diabetes complications but it does not have strong consistency over time and requires HOMA2 indices, which are not routinely available in clinical practice and standard cohort studies. We developed a machine learning (ML) model to classify individuals with type 2 diabetes into Ahlqvist’s subtypes consistently over time.

**Methods:**

Cohort 1 dataset comprised 619 Japanese individuals with type 2 diabetes who were divided into training and test sets for ML models in a 7:3 ratio. Cohort 2 dataset, comprising 597 individuals with type 2 diabetes, was used for external validation. Participants were pre-labelled (T2D_kmeans_) by unsupervised *k*-means clustering based on Ahlqvist’s variables (age at diagnosis, BMI, HbA_1c_, HOMA2-B and HOMA2-IR) to four subtypes: severe insulin-deficient diabetes (SIDD), severe insulin-resistant diabetes (SIRD), mild obesity-related diabetes (MOD) and mild age-related diabetes (MARD). We adopted 15 variables for a multiclass classification random forest (RF) algorithm to predict type 2 diabetes subtypes (T2D_RF15_). The proximity matrix computed by RF was visualised using a uniform manifold approximation and projection. Finally, we used a putative subset with missing insulin-related variables to test the predictive performance of the validation cohort, consistency of subtypes over time and prediction ability of diabetes complications.

**Results:**

T2D_RF15_ demonstrated a 94% accuracy for predicting T2D_kmeans_ type 2 diabetes subtypes (AUCs ≥0.99 and F1 score [an indicator calculated by harmonic mean from precision and recall] ≥0.9) and retained the predictive performance in the external validation cohort (86.3%). T2D_RF15_ showed an accuracy of 82.9% for detecting T2D_kmeans_, also in a putative subset with missing insulin-related variables, when used with an imputation algorithm. In Kaplan–Meier analysis, the diabetes clusters of T2D_RF15_ demonstrated distinct accumulation risks of diabetic retinopathy in SIDD and that of chronic kidney disease in SIRD during a median observation period of 11.6 (4.5–18.3) years, similarly to the subtypes using T2D_kmeans_. The predictive accuracy was improved after excluding individuals with low predictive probability, who were categorised as an ‘undecidable’ cluster. T2D_RF15_, after excluding undecidable individuals, showed higher consistency (100% for SIDD, 68.6% for SIRD, 94.4% for MOD and 97.9% for MARD) than T2D_kmeans_.

**Conclusions/interpretation:**

The new ML model for predicting Ahlqvist’s subtypes of type 2 diabetes has great potential for application in clinical practice and cohort studies because it can classify individuals with missing HOMA2 indices and predict glycaemic control, diabetic complications and treatment outcomes with long-term consistency by using readily available variables. Future studies are needed to assess whether our approach is applicable to research and/or clinical practice in multiethnic populations.

**Graphical Abstract:**

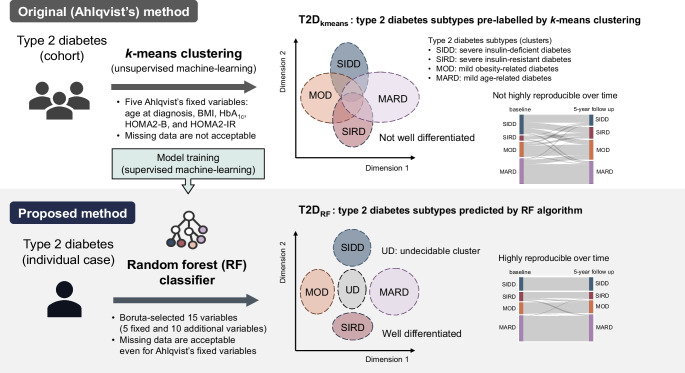

**Supplementary Information:**

The online version of this article (10.1007/s00125-024-06248-8) contains peer-reviewed but unedited supplementary material.


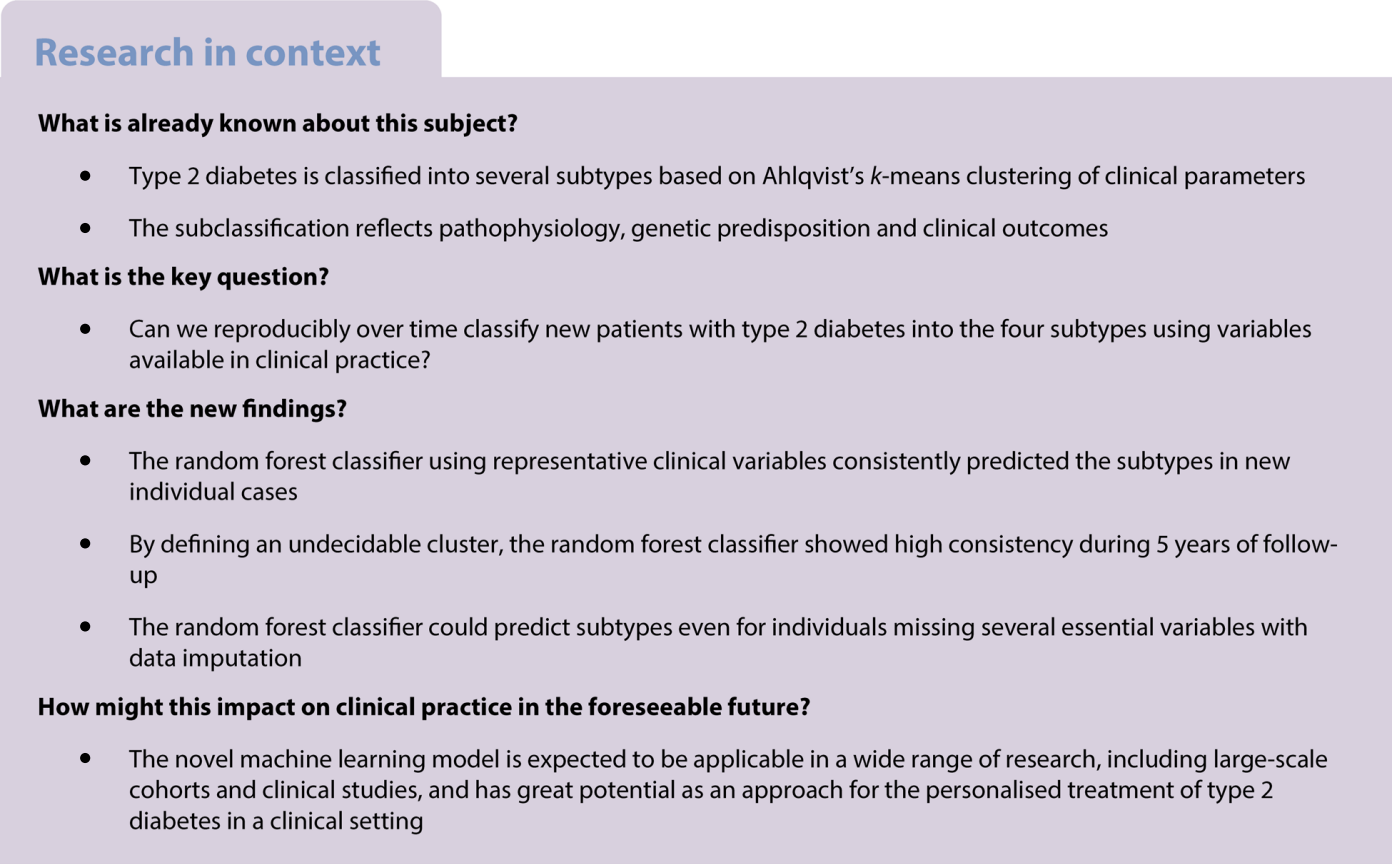


## Introduction

Diabetes mellitus is generally classified into type 1 and type 2 based on its aetiology [1]. Type 1 diabetes is mainly caused by beta cell dysfunction due to autoimmune mechanisms, whereas type 2 diabetes is caused by the heterogeneous influence of insulin resistance and beta cell dysfunction [2]. When choosing a glucose-lowering drug, the decision has recently shifted from being based on side effects and cost-effectiveness to being based on evidence for the prevention of diabetes complications, such as CVD, heart failure and chronic kidney disease (CKD) [3, 4]. However, the pathophysiology, genetic risk and involvement of environmental factors such as diet, physical activity and stress vary widely among individuals with type 2 diabetes [5]. Therefore, a personalised approach that comprehensively considers these factors is crucial [6, 7].

Artificial intelligence (AI), including machine learning (ML), is rapidly being applied to diagnosis, treatment and management in diabetes care and research [8]. Using ML techniques, Ahlqvist et al found five diabetes clusters with different clinical phenotypes and outcomes in a Nordic population: Cluster 1, severe autoimmune diabetes (SAID); Cluster 2, severe insulin-deficient diabetes (SIDD); Cluster 3, severe insulin-resistant diabetes (SIRD); Cluster 4, mild obesity-related diabetes (MOD); and Cluster 5, mild age-related diabetes (MARD) [9]. The SAID cluster resembles type 1 diabetes, whereas the other clusters correspond to type 2 diabetes. These diabetes subtypes have been replicated in cohorts including various ethnic groups in terms of genetic predisposition, glycaemic control, diabetes complications and treatment outcomes [10–15]. This suggests the effectiveness of a personalised approach using the diabetes subtypes [16–18].

However, there are several limitations when applying Ahlqvist’s diabetes clustering in clinical settings and other research. First, the diabetes clustering cannot classify new individuals that are not included in their mother dataset because it depends on the relative positioning of individuals in an entire dataset map [19]. Second, the diabetes clustering cannot be applicable when there are missing fixed variables, HOMA2-B and HOMA2-IR, which represent two key pathogenic mechanisms but are not routinely available in clinical practice and standard cohort studies [20]. For instance, an attempt to replicate the clustering using nine clinical variables, excluding HOMA2 indices, failed to identify Ahlqvist’s clusters [21]. Another study employing C-peptide and HDL-cholesterol instead of HOMA2 indices was unsuccessful in classifying individuals to Ahlqvist’s subtypes [22]. Third, although the diabetes subtypes are theoretically stable over time, a proportion of individuals migrate between subtypes over time [13, 15, 23], limiting the use of this subtyping approach for estimating long-term treatment response and prognosis. As an example, Bello-Chavolla et al reported an AI approach using a self-normalising neural network (SNNN) model [15], showing that proportions of type 2 diabetes clusters were largely different at baseline vs 2 years of follow-up: SIDD 34% vs 16%; SIRD 7% vs 7%; MOD 41% vs 54%; and MARD 18% vs 23% [15].

In this study, an interdisciplinary team of diabetologists and ML specialists aimed to develop an ML model to classify individuals with type 2 diabetes consistently over time into Ahlqvist’s subtypes by minimising the above limitations [9].

## Methods

### Study design and participants

We included participants from two distinct geographical areas in Japan, Fukushima (Cohort 1) and Okinawa (Cohort 2), to target a wide range of genetic backgrounds [24]. The study protocol was approved by the Ethics Committee of the Fukushima Medical University (approval no. REC 2022-028). The sex of participants was determined by self-report.

#### Cohort 1

**Fig. 1:**
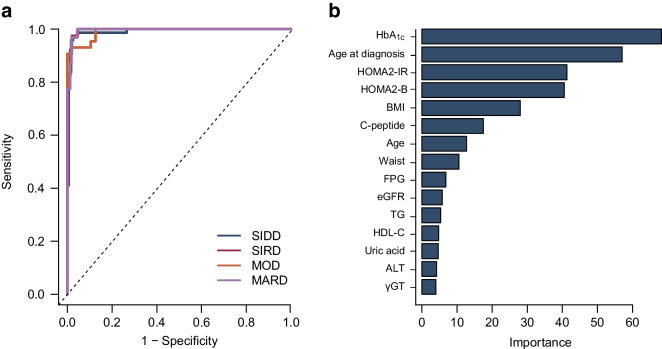
Predictive performance of type 2 diabetes subtypes using an RF algorithm based on 15 features (T2D_RF15_) for estimating T2D_kmeans_ in the test dataset of Cohort 1. (**a**) ROC curve showing the diagnostic performance of T2D_RF15_, the RF model using Boruta-selected 15 features, to predict the T2D_kmeans_. (**b**) Feature importance of Boruta-selected 15 variables fed into T2D_RF15_. ALT, aspartate aminotransferase; FPG, fasting plasma glucose; γGT, γ-glutamyl transferase; HDL-C, HDL-cholesterol; TG, triacylglycerols

The Fukushima Diabetes, Endocrinology, and Metabolism (Fukushima-DEM) cohort was a retrospective and prospective survey of participants with impaired glucose tolerance and diabetes at the Fukushima Medical University to clarify the risk factors for the onset and progression of diabetes and its complications [10]. The flow from registration to dataset construction is shown in electronic supplementary material (ESM) Fig. 1. The participants were recruited between January 2018 and March 2023 and followed up until December 2023. Of the 897 participants, 619 were diagnosed with type 2 diabetes based on the diagnostic criteria described below. Participants without diabetes (*n*=153), with type 1 diabetes (*n*=70), with secondary diabetes (*n*=49) or who had missing clustering variables (*n*=6) were excluded. After labelling with *k*-means clustering, 70% of the total sample was randomly selected for training and the remaining 30% was used for testing.

#### Cohort 2

The Shimajiri Kinsermae Diabetes Care Clinic cohort was a prospective study of individuals with impaired glucose tolerance and diabetes recruited from Okinawa, Japan. The participants were recruited between January 2020 and January 2021. Of the 1253 participants, 597 were diagnosed with type 2 diabetes based on the diagnostic criteria described below (ESM Fig. 1). Participants without diabetes (*n*=248), with type 1 diabetes (*n*=31), with secondary diabetes (*n*=5) or who had missing clustering variables (*n*=372) were excluded. After labelling with *k*-means clustering, the data were used as external validation data for the trained model. A subset with completely missing insulin-related variables (HOMA2-B, HOMA2-IR and C-peptide) was separately created and used as validation data after missing imputation. The need for informed consent in Cohort 2 was waived by the ethics committee because the research did not use identifiable private information and involved no more than minimal risk to the participants. Participants were given the option to decline the use of their personal data based on documents posted on bulletin boards or clinic websites.

### Measurements

Variables such as height, weight, waist circumference and BP of participants in both cohorts were measured during study enrolment and the participants visited the clinic at intervals of 1–3 months. Waist circumference was measured at the level of the umbilicus (cm) in the standing position. Blood samples were collected at baseline in the morning after overnight fasting for ≥10 h and assayed within 1 h using automatic clinical chemical analysers. HOMA2-B and HOMA2-IR were calculated using a HOMA2 calculator (University of Oxford, Oxford, UK) based on fasting plasma glucose and fasting serum C-peptide concentrations measured at baseline [25]. Outliers in the HOMA2 calculator for fasting plasma glucose level (<3 mmol/l or >25 mmol/l) and C-peptide level (<0.2 nmol/l or >3.5 nmol/l) were capped to lower or upper limit values. We calculated the eGFR using the Japanese formula [26].

### Definitions

The criteria for diagnosing diabetes were as follows: fasting plasma glucose level ≥7.0 mmol/l; random plasma glucose level ≥11.1 mmol/l; HbA_1c_ level ≥48 mmol/mol (6.5%); or regular use of glucose-lowering drugs. At least one previously confirmed positive result for an islet-associated autoantibody is indicative of type 1 diabetes. The severity of diabetic retinopathy was determined based on fundus photography by qualified ophthalmologists. According to the modified international clinical diabetic retinopathy severity scales [27], we classified participants into the following three groups: no diabetic retinopathy; non-proliferative diabetic retinopathy; and proliferative diabetic retinopathy. Where severity in the right or left eye was different, more severe staging was performed. If either non-proliferative or proliferative diabetic retinopathy was present, diabetic retinopathy was diagnosed. CKD was defined as an eGFR <60 ml/min per 1.73 m^2^ for more than 90 days, and proteinuria was defined as albuminuria ≥30 mg/g creatinine. Coronary artery disease was defined using the ICD-10 codes I20–21, I24, I251 or I253–259 (https://icd.who.int/browse10/2019/en).

### ML algorithm

#### The *k*-means clustering and random forest classifier

The *k*-means clustering was applied to create the true labels (type 2 diabetes subtypes pre-labelled by *k*-means clustering [T2D_kmeans_]) for an ML model in the two cohorts. Using the fpc R package (version 2.2-11, https://cran.r-project.org/web/packages/fpc/index.html), *k*-means clustering was performed 1000 times (*k*=4), following the method of Ahlqvist et al [9]. Ahlqvist’s variables (age at diagnosis, BMI, HbA_1c_, HOMA2-B and HOMA2-IR) were used for the cluster analysis. To minimise the effects of sex, men and women were clustered separately. The stability of clustering was assessed using the Jaccard index after 2000× resampling of the dataset [28].

An ML model was then constructed to predict type 2 diabetes subtypes from new data using random forest (RF), a supervised approach. The RF classifier is an efficient algorithm that uses a subset of randomly selected training samples and variables to generate multiple decision trees [29] and has consistently outperformed other classifiers [30]. Furthermore, the RF classifier is less affected by multicollinearity in high-dimensional data, is faster and less susceptible to overtraining, and can calculate the importance of features [31]. Cohort 1 was used to train an RF multiclass classification model that predicted type 2 diabetes subtypes (randomForest R package version 4.7-1.1, https://cran.r-project.org/web/packages/randomForest/index.html). The parameters of the RF algorithm, such as the random sample size, number of trees, minimum number of termination nodes and maximum number of termination nodes, were tuned to improve the prediction performance [32].

We trained an RF model (type 2 diabetes subtypes predicted by RF algorithm based on five variables [T2D_RF5_]), based on Ahlqvist’s variables age at diagnosis, BMI, HbA_1c_, HOMA2-B and HOMA2-IR, to assess its accuracy for estimating the true labels (T2D_kmeans_). To address potential missing Ahlqvist’s variables, especially insulin-related ones, an extended RF model (type 2 diabetes subtypes predicted by RF algorithm based on 15 variables [T2D_RF15_]) was constructed to predict type 2 diabetes subtypes based on 15 variables. We made T2D_RF15_ by applying the Boruta algorithm to select 15 important features out of an initial 25, which were chosen based on their availability in clinical settings. The importance of the features and the predictive metrics of T2D_RF5_ and T2D_RF15_ for T2D_kmeans_ subtypes were calculated.

The RF algorithm creates a proximity matrix as a byproduct. The proximity matrix is defined as the frequency with which two cases are classified into the same leaf node in the decision tree of the established model and represents the degree of similarity between samples [33]. Uniform manifold approximation and Projection (UMAP) was used to embed this matrix in two dimensions for visualisation of individual prediction probabilities calculated by T2D_RF15_.

#### RF prediction in a dataset with missing variables

We aimed to make the T2D_RF15_ model applicable to individuals who are missing insulin-related variables. First, we intentionally deleted insulin-related variables in Cohort 2 and then imputed these missing values using an RF regression analysis (ESM Fig. 1). Second, the Cohort 2 individuals imputed were classified by T2D_RF15_. Third, to evaluate the importance of variables, we determined the prediction accuracy of T2D_RF15_ for labelling by T2D_kmeans_ when variables were omitted step-wise for three insulin-related variables and the others. Proportions of undecidable individuals were also determined. Fourth, the performance of T2D_RF15_ was further evaluated using precision (% of data that actually belonged to the predicted clusters), recall (% of data that each RF model correctly predicts belongs to that cluster: sensitivity), F1-score (an indicator calculated by harmonic mean from precision and recall) and AUC for the receiver operating characteristic (ROC) curve for each subtype.

Kaplan–Meier curves for the cumulative incidence of retinopathy, CKD (eGFR <60 ml/min per 1.73 m^2^) and coronary artery disease in the type 2 diabetes subtypes were predicted by T2D_RF15_ on the putative dataset in Cohort 1.

### Consistency over time

The consistency over time of subtype classification in four models, T2D_kmeans_, SNNN model [15], T2D_RF15_ and T2D_RF15_ with missing insulin-related variables, was assessed by migration patterns at baseline and 5 year follow-up in Sankey diagrams. The consistency over time was assessed by the percentage of participants whose subtype classification did not change between baseline and 5 year follow-up.

### Statistical analysis

Continuous and parametric values are presented as mean ± SD, and non-parametric values are presented as median (first quartile–third quartile). Group differences were analysed using one-way ANOVA or the Kruskal–Wallis test. Categorical values are presented as percentages, and group differences were analysed using the χ^2^ test.

Survival analysis for the cumulative incidence of diabetes complications in Cohort 1 was performed using the Kaplan–Meier method for T2D_RF15_ clusters. HRs and 95% CIs were subsequently calculated using the Cox proportional hazards model. Missing values in the training data (rate is shown in Table 1) were imputed using the Multivariate Imputation by Chained Equations (MICE) algorithm [34]. Ten complete datasets were generated through this imputation process. The estimated values from each imputed dataset were integrated using Rubin’s rule [35].

**Table 1:** Clinical characteristics of study participants at baseline in Cohort 1 stratified by type 2 diabetes subtypes predicted by RF classifier trained using 15 selected features (T2D_RF15_)

Clinical feature	Missing rate (%)	Overall (*n*=619)	T2D subtypes predicted by RF classifier (T2D_RF15_)					*p* value
			SIDD (*n*=116; 18.7%)	SIRD (*n*=90; 14.5%)	MOD (*n*=109; 17.6%)	MARD (*n*=216; 34.9%)	Undecidable (*n*=88; 14.2%)	
Demographic characteristics								
Female, *n* (%)	0	280 (45)	43 (37)	44 (49)	58 (53)	101 (47)	34 (39)	0.086
Age, years	0	69 ± 13	68 ± 12	63 ± 14	60 ± 13	76 ± 7	68 ± 12	<0.001
Age at diagnosis, years	0	51 ± 12	44 ± 10	48 ± 12	41 ± 7	60 ± 7	50 ± 12	<0.001
Duration of diabetes, years	0	18 ± 9	24 ± 11	14 ± 8	19 ± 9	16 ± 8	19 ± 11	<0.001
Current smoker, *n* (%)	0	95 (15)	18 (16)	21 (23)	18 (17)	27 (13)	11 (13)	0.169
Alcohol use, *n* (%)	0	181 (29)	30 (26)	27 (30)	30 (28)	65 (30)	29 (33)	0.830
Anthropometric data								
BMI, kg/m^2^	0	26.5 ± 6.4	24.5 ± 4.7	32.9 ± 8.5	30.7 ± 5.3	22.5 ± 2.7	27.1 ± 5.2	<0.001
Waist circumference, cm	1.6	92 ± 15	90 ± 13	106 ± 17	100 ± 12	84 ± 9	95 ± 13	<0.001
Systolic BP, mmHg	0	132 ± 18	128 ± 18	132 ± 18	134 ± 19	131 ± 18	133 ± 16	0.116
Diastolic BP, mmHg	0	73 ± 12	72 ± 12	75 ± 13	77 ± 11	71 ± 11	74 ± 12	0.006
Laboratory measurements								
Fasting plasma glucose, mmol/l	0	7.7 ± 2.0	8.9 ± 2.4	7.3 ± 1.6	7.3 ± 2.0	7.4 ± 1.5	7.9 ± 2.4	<0.001
HbA_1c_, mmol/mol	0	54 ± 11	72 ± 11	49 ± 6	49 ± 6	49 ± 5	53 ± 2	<0.001
HbA_1c_, %	0	7.1 ± 1.1	8.7 ± 1.0	6.6 ± 0.5	6.7 ± 0.5	6.6 ± 0.5	7.0 ± 0.8	<0.001
Fasting serum C-peptide, nmol/l	0	0.80 ± 0.51	0.54 ± 0.29	1.59 ± 0.65	0.73 ± 0.29	0.64 ± 0.28	0.80 ± 0.42	<0.001
HOMA2-B	0	67.7 ± 36.9	37.4 ± 11.2	132.2 ± 39.9	69.6 ± 18.3	56.0 ± 15.8	68.2 ± 30.9	<0.001
HOMA2-IR	0	1.84 ± 1.08	1.31 ± 0.53	3.86 ± 0.97	1.59 ± 0.47	1.39 ± 0.48	1.87 ± 0.93	<0.001
Triacylglycerols, mmol/l	0	1.2 (0.8–1.3)	1.2 (0.9–2.0)	1.7 (1.3–2.3)	1.3 (0.9–1.8)	1.0 (0.7–1.4)	1.3 (0.9–1.9)	<0.001
HDL-cholesterol, mmol/l	0	1.4 ± 0.4	1.4 ± 0.4	1.3 ± 0.3	1.4 ± 0.3	1.5 ± 0.4	1.4 ± 0.3	<0.001
LDL-cholesterol, mmol/l	0	2.7 ± 0.8	2.6 ± 0.8	2.7 ± 0.7	2.7 ± 0.7	2.6 ± 0.8	2.8 ± 0.9	0.597
AST, U/l	0	21 (17–28)	21 (17–28)	23 (17–36)	20 (16–28)	21 (17–26)	21 (17–28)	0.109
ALT, U/l	0	19 (14–30)	21 (15–31)	26 (15–47)	20 (15–35)	17 (12–24)	18 (15–27)	<0.001
γGT, U/l	0	25 (17–42)	26 (18–44)	38 (23–59)	24 (16–36)	22 (17–35)	26 (17–39)	<0.001
eGFR, ml/min per 1.73 m^2^	0	63 ± 19	66 ± 21	56 ± 22	70 ± 19	62 ± 16	61 ± 19	<0.001
Uric acid, μmol/l	0.2	321 ± 78	308 ± 81	359 ± 75	315 ± 70	308 ± 74	338 ± 83	<0001
White blood cell, 10^3^/μl	0	6.3 ± 1.9	6.4 ± 1.9	6.7 ± 1.9	6.5 ± 1.7	5.7 ± 1.9	6.6 ± 2.1	<0.001
Haemoglobin, g/l	0	136 ± 18	139 ± 17	141 ± 20	138 ± 17	132 ± 17	136 ± 18	<0.001
Platelets, 10^4^/μl	0	22.2 ± 6.6	22.2 ± 5.7	21.9 ± 6.1	23.2 ± 6.7	21.3 ± 7.0	23.2 ± 6.7	0.078
Albuminuria, mg/gCr	2.6	21 (8–86)	28 (8–155)	35 (10–165)	18 (7–69)	16 (7–44)	28 (8–153)	0.009
Glucose-lowering drugs, *n* (%)								
Sulfonylurea	0	53 (9)	18 (16)	4 (4)	11 (10)	16 (7)	4 (5)	0.020
Metformin	0	291 (47)	69 (60)	41 (40)	67 (62)	73 (34)	43 (49)	<0.001
DPP-4 inhibitor	0	352 (57)	65 (56)	38 (42)	73 (67)	132 (61)	44 (50)	0.003
SGLT2 inhibitor	0	157 (25)	41 (35)	28 (31)	30 (28)	33 (15)	25 (28)	<0.001
GLP-1 receptor agonist	0	64 (10)	20 (17)	19 (21)	11 (10)	3 (1)	11 (13)	<0.001
Insulin	0	184 (30)	72 (62)	8 (9)	28 (26)	50 (23)	26 (30)	<0.001

Values are presented as mean ± SD, median (IQR) or *n* (%)

*p* values were obtained by one-way ANOVA, Kruskal–Wallis test or χ^2^ test

AST, aspartate aminotransferase; ALT, alanine aminotransferase; DPP-4, dipeptidyl peptidase-4; gCr, g of creatinine; GLP-1, glucagon-like peptide-1; γGT, γ-glutamyl transpeptidase; SGLT, sodium–glucose cotransporter 2; T2D, type 2 diabetes

A *p* value of <0.05 indicated statistical significance. All statistical analyses were performed using R version 4.3.1 (https://www.r-project.org/).

## Results

### *k*-means cluster distribution and characteristics

In Cohort 1, the training dataset was pre-labelled (T2D_kmeans_) for the type 2 diabetes subtype (SIDD, SIRD, MOD or MARD) using unsupervised *k*-means clustering. The cluster centre coordinates stratified by sex are shown in ESM Table 1. The Jaccard index (min–max) was 0.76–0.90 for women and 0.79–0.93 for men. As shown in ESM Table 2, the following characteristics were noted: the SIDD cluster had low HOMA2-B and high HbA_1c_ levels; the SIRD cluster had high BMI, HOMA2-B and HOMA-IR; the MOD cluster had a younger age at diagnosis and high BMI; and MARD was the most common cluster and had the oldest age at diagnosis. The characteristics of T2D_kmeans_ were similar to those described by Ahlqvist et al [9].

### Type 2 diabetes subtypes using RF algorithm

**Fig. 2:**
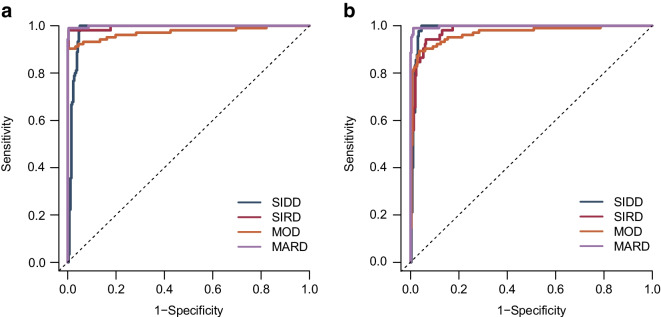
Predictive performance of type 2 diabetes subtypes using an RF algorithm based on 15 features (T2D_RF15_) for estimating T2D_kmeans_ in the external validation dataset of Cohort 2. ROC curve showing the diagnostic ability of T2D_RF15_ to predict the subtypes pre-labelled by *k*-means clustering (T2D_kmeans_) were calculated in original Cohort 2 dataset (**a**) and in a putative Cohort 2 dataset with missing insulin-related variables (**b**)

The model performance in T2D_RF5_, T2D_RF15_ and T2D_RF25_ was assessed by metrics for predicting T2D_kmeans_ (ESM Table 3). For T2D_RF5_, the overall prediction performance was 94.0%**,** and AUC values for subtypes are 99.5% for SIDD, 98.4% for SIRD, 99.1% for MOD and 99.0% for MARD. For T2D_RF15_, the overall prediction performance was robust, achieving 94.1% of AUC (Fig. 1a), and the prediction accuracy for all subtypes was validated with high precision, recall values and F1 scores≥0.9 (ESM Table 3). Among the 15 variables, C-peptide level, age and waist circumference, besides Ahlqvist’s five variables, were the most important for T2D_RF15_ subtype prediction (Fig. 1b). The order of importance of variables varied considerably between subtypes (ESM Fig. 2).

### External validation of the predicting model

The validity of the RF multiclass classification model trained with the 15 features was evaluated in Cohort 2 to confirm its applicability to external data. The ROC curves comparing T2D_kmeans_ and T2D_RF15_ are shown in Fig. 2a. The overall accuracy was 86.3%, and the model performance was retained when applied to the external cohort. The detailed consistency indices are shown in ESM Table 3.

### Classification approach for individuals with missing clustering variables

**Fig. 3:**
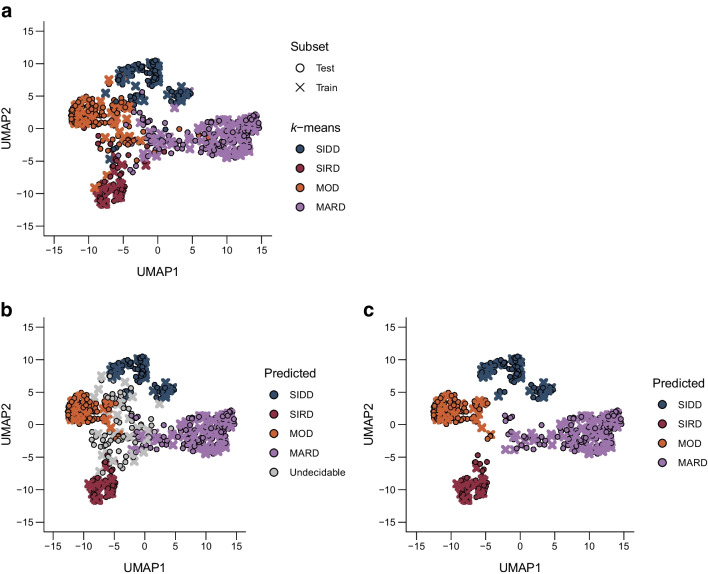
Proximity matrix representing the similarity between participants calculated using the RF. (**a**) Two-dimensional visualisation of the proximity matrix between all participants included in the training and test data. Colours indicate differences in subtype assignment using *k*-means clustering (T2D_kmeans_). (**b**) Proximity matrix with embedded labels for type 2 diabetes subtypes predicted by the RF algorithm based on 15 variables (T2D_RF15_). Participants with low predictive probability were newly defined as the undecidable cluster. (**c**) Proximity matrix with T2D_RF15_ labels embedded after excluding participants in the undecidable cluster; the remaining participants could be clearly divided into four clusters

**Fig. 4:**
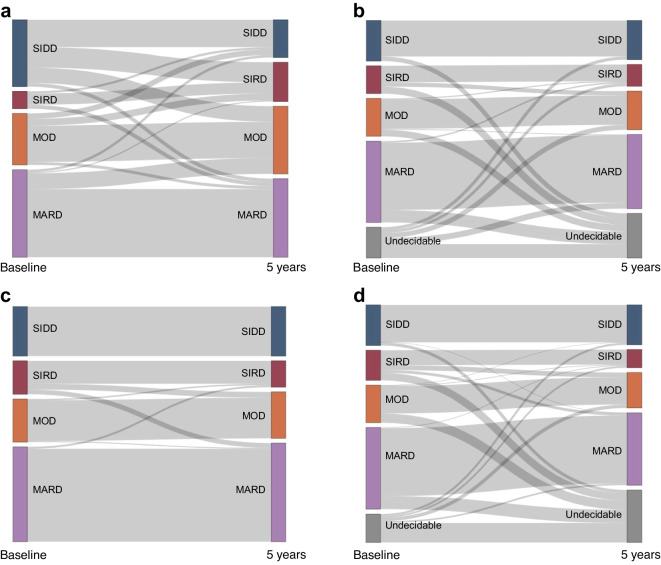
Sankey diagram showing the subtype redistribution and migration pattern of the study participants in Cohort 1 from baseline to 5 year follow-up. (**a**) Type 2 diabetes subtypes labelled by *k*-means clustering (T2D_kmeans_). (**b**) Type 2 diabetes subtypes predicted by an RF algorithm based on 15 variables (T2D_RF15_). (**c**) Migration pattern of T2D_RF15_ excluding the undecidable cluster. (**d**) Type 2 diabetes subtypes predicted by an RF algorithm based on 15 variables from the dataset where insulin-related variables have been imputed (T2D_RF15_)

Correlations of the insulin-related variables, C-peptide, HOMA2-B and HOMA2-IR, between observed and predicted values showed strong correlations in Cohort 2 with missing insulin-related variables (*R*^2^=0.83–0.92) (ESM Fig. 3 a–c). The mean absolute differences of these variables were small and normally distributed, suggesting a relatively small impact of imputing the insulin-related variables on subtype predictions (ESM Fig. 3 d–f). The predictive performance (ROC) by T2D_RF15_, including imputed insulin-related variables, is shown in Fig. 2b. The overall prediction performance of T2D_RF15_ was 82.9%, and AUC values for the diabetes subtypes were 97.4% for SIDD, 96.4% for SIRD, 93.7% for MOD and 97.6% for MARD (ESM Table 3). The impact of missing variables on classification metrics of T2D_RF15_ is shown in ESM Fig. 4. When omitting variables, the prediction accuracy of T2D_RF15_ did not change in individuals until a decrease was seen when age and BMI were omitted from the insulin-related variables (ESM Fig. 4a). Similarly, the proportion of undecidable individuals did not alter age and BMI were omitted (ESM Fig. 4b). The classification metrics per cluster also did not change until age and BMI were omitted (Fig. 4c, numbers 12 and 13 on *x*-axis) but the declines of values was more rapid in SIRD and MOD than in SIDD and MARD (ESM Fig. 4c).

### Evaluating consistency over time and clarity of type 2 diabetes subtype classification

**Fig. 5:**
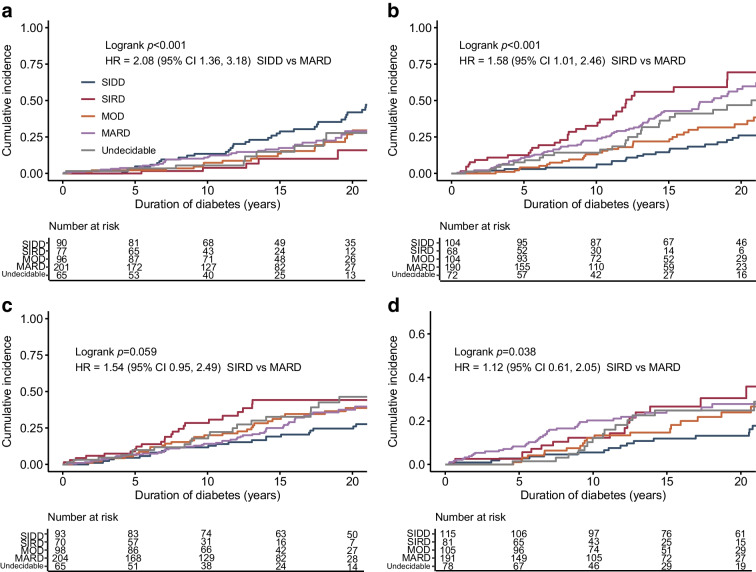
Kaplan–Meier curves for the cumulative incidence of retinopathy (**a**), CKD (eGFR <60 ml/min per 1.73 m^2^) (**b**), proteinuria (**c**) and coronary artery disease (**d**) in type 2 diabetes subtypes predicted by RF based on 15 variables (T2D_RF15_) in the putative dataset in Cohort 1 with missing insulin-related variables

The similarities between participants was visualised by UMAP, using the proximity matrix calculated by RF, and colour-coded with T2D_kmeans_ (Fig. 3a) and T2D_RF15_ (Fig. 3b). When the individual predictive probabilities computed in the RF were embedded in the proximity matrix, participants with low predictive probabilities were located in the boundary regions of the subtypes (ESM Fig. 5). The data with a predictive probability of less than 0.6 were defined and relabelled as an ‘undecidable cluster’ to minimise uncertainty in the T2D_RF15_ model (Fig. 3b). This group of data (accounting for 14.2% of all participants) was located in the boundary region; after excluding them, the data were clearly divided into four clusters, showing high predictive reliability (Fig. 3c). After excluding the undecidable cluster, the clinical characteristics of T2D_RF15_ subtypes for SIDD, SIRD, MOD and MARD (Table 1) were almost identical to those of T2D_kmeans_ reported previously [10]. In contrast, the undecidable cluster showed no distinctive clinical characteristics. For example, in this type, the percentage of female sex was as low as in SIDD; age was higher than in SIRD and MOD but lower than in MARD; BMI was higher than in SIDD and MARD but lower than in SIRD and MOD; and HOMA2-IR was higher than in SIDD and MARD but lower than in SIDD and MARD.

We tested the consistency of subtype classification at baseline and after 5 years in T2D_kmeans_, SNNN, T2D_RF15_ and T2D_RF15_ with missing insulin-related variables. T2D_kmeans_ showed low consistency (Fig. 4a; 58.9% for SIDD, 53.8% for SIRD, 70.6% for MOD and 77.8% for MARD). SNNN also showed low consistency (ESM Fig. 6). In contrast, T2D_RF15_, after excluding the undecidable cluster, showed higher consistency (Fig. 4b,c; 100% for SIDD, 68.6% for SIRD, 94.4% for MOD and 97.9% for MARD) than those of T2D_kmeans_. The mean consistency for four type 2 diabetes subtypes between baseline and 5 years of follow-up was 96.2%, compared with 49.5% in the undecidable cluster. T2D_RF15_ with missing insulin-related variables also showed a high consistency (mean 94.1%, except for the undecidable cluster, Fig. 4d).

### Survival analysis of diabetes complications

To test whether T2D_RF15_ could predict clinical outcomes, Kaplan–Meier analysis of diabetes complications was performed in a putative dataset in Cohort 2 with missing insulin-related variables (Fig. 5). The median observation period was 11.6 (IQR 4.5–18.3) years. The cumulative incidence of diabetic retinopathy and CKD differed among the diabetes subtypes. After adjusting for baseline age and sex, the risk for diabetic retinopathy was higher in the SIDD cluster than in the MARD cluster (HR 2.08 [95% CI 1.36, 3.18], *p*<0.001). Similarly, the risk of CKD was higher in the SIRD cluster than in MARD (HR 1.58 [95% CI 1.01, 2.46], *p*<0.001). These findings were consistent with those of previous reports [9, 10] that had determined the subtypes using *k*-means clustering (T2D_kmeans_). Namely, the risk of CKD was higher in the SIRD cluster of T2D_kmeans_ than in MARD (the age- and sex-adjusted HR 2.41 [95% CI 2.08, 2.79], *p*<0.0001 in the Nordic population [9]; HR 1.60 [95% CI 1.03, 2.47], *p*=0.035 in our Japanese population [10]). The risk of diabetic retinopathy was higher in the SIDD cluster of T2D_kmeans_ than in MARD (the age- and sex-adjusted HR 1.33 [1.15, 1.54], *p*<0.0001 in the Nordic population [9]; HR 1.78 [95% CI 1.30, 2.43], *p*<0.001 in our Japanese population [10]). Meanwhile, the undecidable cluster had an intermediate risk for all complications (Fig. 5). Namely, the Kaplan–Meier curves for the cumulative incidence of retinopathy, CKD and coronary artery disease in the undecidable cluster lay between the highest and lowest curves (Fig. 5).

## Discussion

We developed an ML model that easily and consistently classifies individuals with type 2 diabetes into Ahlqvist’s subtypes by minimising the disadvantages. Three main improvements were achieved: (1) our ML model employed RF classifiers instead of original *k*-means [19], which enabled us to predict Ahlqvist subtypes for new individuals that were not included in the mother dataset; (2) by integrating imputation algorithms, the RF classifier was able to accurately predict type 2 diabetes subtypes even for individuals with missing HOMA2-B and HOMA2-IR [20]; and (3) by defining an undecidable cluster, the RF classifier achieved high consistency during 5 years of follow-up in the subtype classification. This new ML model has great potential for clinical practice and cohort studies because it can classify individuals newly diagnosed with type 2 diabetes into Ahlqvist’s subtypes using readily available variables.

Our ML model enables us to classify individuals into Ahlqvist’s subtypes by employing an RF classifier. Owing to its ease of implementation and low computational complexity, *k*-means clustering, an unsupervised ML algorithm, is most frequently used among several methods for AI subtyping [36]. Actually, Ahlqvist’s *k*-means clustering based on five fixed variables [37], including age at onset, BMI, HbA_1c_, HOMA2-B and HOMA2-IR, is the most extensively studied in diabetes research [9–12, 14]. However, the *k*-means clustering cannot classify new individual cases not included in their mother dataset because it depends on the positioning of cases in an entire dataset map [19]. Previously, one of our team found that RF-based ML algorithms are useful for risk stratification beyond conventional classifications and are applicable case by case in people with ovarian cancer [38] or heart failure [39]. In this study, we similarly created a novel ML model based on RF and developed a method to determine Ahlqvist’s subtype on a case-by-case basis.

By integrating imputation algorithms, the RF classifier was able to accurately predict type 2 diabetes subtypes even for individuals with missing insulin-related variables. As discussed above, the diabetes clustering cannot be applied when the fixed variables HOMA2-B and HOMA2-IR are missing [21, 22]. C-peptide levels, which are used to calculate the HOMA2 indices, are not routinely measured in people with diabetes in clinical practice and in standard cohort studies, usually due to the cost. Our RF classifier could predict diabetes subtypes, even when C-peptide was missing, by imputing with high consistency. To our knowledge, this study for the first time shows that the RF classifier can predict diabetes subtypes even when insulin-related variables are missing.

Our ML model showed long-term consistency in all four diabetes clusters. Consistency over time of previous AI models in determining type 2 diabetes subtypes has been limited. Bello-Chavolla et al reported an approach for classifying diabetes subtypes using an SNNN model [15]. Since subtype consistency during follow-up with this approach was low, they considered that diabetes subtypes are changeable and should be reassessed periodically to understand the trajectories and risks of diabetes complications [15]. However, when applying their SNNN model to our participants in Cohort 1, the consistency of the subtypes was also low (Fig. 3b): the SNNN model demonstrated an overall accuracy of 69% but was particularly low for the SIDD (36.4%) and SIRD (16.3%) clusters. The difference in consistency over time between RF classification and SNNN in the same population suggests that the diabetes subtype is simply not correctly determined rather than changeable. The diabetes subtype should be consistent in an individual over years of long clinical course in terms of genetic risk [40], molecular mechanisms [41] and complication risk [9, 10]. We achieved excellent long-term consistency in subtype classification by excluding an undecidable cluster in all four diabetes subtypes. Given that previous studies on diabetes subtypes have used ‘hard’ clustering methods such as *k*-means, which forcefully assigns samples at boundaries of clusters to either cluster, we a priori hypothesised that ‘hard’ clustering leads to lower consistency in diabetes subtyping. Therefore, we employed the idea of grouping samples with low prediction probability by the RF classifier (i.e. populations with uncertainty about which subtype they belong to) as a single ‘undecidable’ cluster rather than forcing their assignation to a subtype. This is a clinically acceptable approach, given that BMI and HbA_1c_ often fluctuate during treatment and are inappropriate for inclusion in the subtype prediction. Considering this undecidable cluster, little migration among subtypes occurred after the 5 year follow-up; thereby high consistency was achieved (well differentiated). Individuals in the undecidable cluster had unclear diabetes characteristics and a non-typical course of diabetes complications for the diabetes subtypes, and approximately half of them moved to different subtypes after 5 years (Table 1, Fig. 4b, c).

This study had several limitations. First, the sample size of the training dataset is relatively small. Second, because this study was conducted only in the Japanese population the results cannot be generalised, thereby limiting applicability to other ancestral populations. We tested consistency by recruiting two Japanese cohorts with diverse genetic predispositions. However, future studies are further needed to assess whether our approach is applicable to multiethnic populations. Additionally, whilst the study sample is broadly representative of general demographic distribution of the Japanese population with diabetes in terms of sex, age and socioeconomic factors, the potential limitations and biases of these factors should still be considered when interpreting the results. Third, because some study participants were enrolled after the start of diabetes treatment rather than at the onset of diabetes, the variables used for clustering and prediction could have been affected at least partly by lifestyle interventions and medications the participants received before study enrolment. Fourth, the reasons for group migration and changes in clinical variables in the undecidable cluster are yet to be determined. This undecidable cluster was atypical, with no clear clinical features (Table 1). In the future, the respective characteristics (i.e. clinical features and genetic predisposition) of individuals moving between clusters and of undecidable groups need to be clarified.

In conclusion, we developed a novel ML model for type 2 diabetes subtypes. The new RF-based model for predicting Ahlqvist’s subtypes of type 2 diabetes has great potential for application in a wide range of research, including large-scale cohorts and clinical studies, because it can classify individuals with missing HOMA2 indices and predict glycaemic control, diabetes complications and treatment outcomes with long-term consistency by using readily available variables. Future studies are needed to assess whether our approach is applicable to research and/or clinical practice in multiethnic populations.

## Supplementary Information

Below is the link to the electronic supplementary material.

ESM (PDF 1008 KB)

## Data Availability

The most relevant data generated or analysed during this study are included in this manuscript and the ESM. Further datasets generated during the current study are available from the corresponding author upon reasonable request.

## Abbreviations

AI: Artificial intelligence
CKD: Chronic kidney disease
MARD: Mild age-related diabetes
ML: Machine learning
MOD: Mild obesity-related diabetes
RF: Random forest
ROC: Receiver operating characteristic
SAID: Severe autoimmune diabetes
SIDD: Severe insulin-deficiency diabetes
SIRD: Severe insulin-resistant diabetes
SNNN: Self-normalising neural network
T2D_kmeans_: Type 2 diabetes subtypes pre-labelled by *k*-means clustering
T2D_RF5_: Type 2 diabetes subtypes predicted by RF algorithm based on five variables
T2D_RF15_: Type 2 diabetes subtypes predicted by RF algorithm based on 15 variables
T2D_RF25_: Type 2 diabetes subtypes predicted by RF algorithm based on 25 variables
UMAP: Uniform manifold approximation and projection
