# Supplementary material for: Machine learning-based reproducible prediction of type 2 diabetes subtypes
Source: Diabetologia. 2024 Aug 21;67(11):2446–58. doi: 10.1007/s00125-024-06248-8 (PMC11519166; doi:10.1007/s00125-024-06248-8)
Supplement: Supplementary file 1 — ESM (PDF 1008 KB) [file 125_2024_6248_MOESM1_ESM.pdf]

**Supplementary materials for**

# Machine learning-based reproducible prediction of type 2 diabetes subtypes

*Diabetologia*

Hayato Tanabe[1,2], Masahiro Sato[1], Akimitsu Miyake[3], Yoshinori Shimajiri[4], Takafumi Ojima[3,5], Akira Narita[6], Haruka Saito[1], Kenichi Tanaka[7], Hiroaki Masuzaki[8], Junichiro J Kazama[7], Hideki Katagiri[2], Gen Tamiya[3,6], Eiryo Kawakami[9,10], and Michio Shimabukuro[1]

**Corresponding authors**

Eiryo Kawakami

Michio Shimabukuro

1. Department of Diabetes, Endocrinology, and Metabolism, Fukushima Medical University School of Medicine, Fukushima, Japan.
2. Department of Diabetes, Metabolism and Endocrinology, Tohoku University Graduate School of Medicine, Miyagi, Japan.
3. Department of AI and Innovative Medicine, Tohoku University School of Medicine, Miyagi, Japan.
4. Shimajiri Kinsermae Diabetes Care Clinic, Okinawa, Japan.
5. Department of Statistical Genetics, Osaka University Graduate School of Medicine, Osaka, Japan
6. Tohoku Medical Megabank Organization, Tohoku University, Miyagi, Japan.
7. Department of Nephrology and Hypertension, Fukushima Medical University School of Medicine, Fukushima, Japan.
8. Division of Endocrinology and Metabolism, Second Department of Internal Medicine, University of the Ryukyus Graduate School of Medicine, Okinawa, Japan
9. Department of Artificial Intelligence Medicine, Graduate School of Medicine, Chiba University, Chiba, Japan.
10. Advanced Data Science Project, RIKEN Information R&D and Strategy Headquarters, RIKEN, Yokohama, Japan.

**List of Tables**

ESM Table 1 Cluster center coordinates

ESM Table 2 Clinical characteristics of the study participants in the Cohort 1 stratified by *k*-means clustering

ESM Table 3 Classification metrics for type 2 diabetes subtypes predicted using random forest algorithm

**List of Figures**

ESM Fig.1 Enrollment flow chart of study participants

ESM Fig.2 Important variables selected for each type 2 diabetes subtype using the Boruta algorithm

ESM Fig.3 Profiles of predicted insulin-related variables imputed using the random forest regression algorithm in putative Cohort 2 with missing insulin-related variables

ESM Fig.4 Impact of missing variables on classification metrics in type 2 diabetes random forest (RF) algorithm based on 15 variables ($T2D_{RF15}$)

ESM Fig.5 Two dimensional (2D) visualisation of the proximity matrix with embedded individual prediction probabilities calculated by random forest (RF) algorithm based on 15 variables ($T2D_{RF15}$)

ESM Fig.6 Migration pattern of type 2 diabetes subtype predicted by the self-normalising neural network algorithm (reported by Bello-Chavolla, et al) from baseline to 5-year follow-up for study participants in Cohort 1

**ESM Table 1 Cluster center coordinates**

| | T2D subtypes | Age at diagnosis, years | BMI, kg/m$^2$ | HbA$_{1c}$, mmol/mol (%) | HOMA2-B | HOMA2-IR |
|---|---|---|---|---|---|---|
| Female | SIDD | -0.37909 | -0.27518 | 1.77256 | -0.85613 | -0.46674 |
| | | 46.0 | 24.7 | 74.4 (9.0) | 36.1 | 1.34 |
| | SIRD | -0.26906 | 1.34582 | -0.47450 | 1.72876 | 1.57387 |
| | | 47.3 | 35.2 | 49.5 (6.7) | 132.0 | 3.58 |
| | MOD | -0.79609 | 0.78837 | -0.38662 | -0.02147 | -0.20137 |
| | | 40.9 | 31.6 | 48.5 (6.6) | 67.2 | 1.64 |
| | MARD | 0.69278 | -0.57168 | -0.44266 | -0.22896 | -0.41086 |
| | | 58.9 | 22.8 | 48.9 (6.6) | 59.5 | 1.40 |
| Male | SIDD | -0.66225 | -0.32444 | 1.34558 | -0.74859 | -0.48178 |
| | | 42.6 | 24.4 | 69.4 (8.5) | 39.7 | 1.32 |
| | SIRD | 0.08240 | 0.54675 | -0.37660 | 1.61096 | 1.95056 |
| | | 51.6 | 30.0 | 49.7 (6.7) | 124.5 | 3.82 |
| | MOD | -0.68481 | 0.40123 | -0.31534 | 0.03824 | -0.14037 |
| | | 42.3 | 29.1 | 50.4 (6.8) | 69.5 | 1.69 |
| | MARD | 0.84944 | -0.59492 | -0.36145 | -0.36832 | -0.39919 |
| | | 60.8 | 22.7 | 49.8 (6.7) | 54.7 | 1.41 |

The upper rows of the table represent the coordinates of the cluster centers after normalisation, while the lower rows display the raw values. MARD, mild age-related diabetes; MOD, mild obesity-related diabetes; SIDD, severe insulin-deficient diabetes; SIRD, severe insulin-resistant diabetes; T2D, type 2 diabetes.

**ESM Table 2 Clinical characteristics of the study participants in the Cohort 1 stratified by *k*-means clustering**

| Clinical features | Overall | T2D subtypes stratified by *k*-means clustering | | | | *p* value |
|---|---|---|---|---|---|---|
| | | SIDD | SIRD | MOD | MARD | |
| | *n* =619 | *n* =129 (20.8%) | *n* =104 (16.8%) | *n* =143 (23.1%) | *n* =243 (39.3%) | |
| Demographic characteristics | | | | | | |
| Female, *n* (%) | 280 (45) | 42 (33) | 51 (47) | 73 (51) | 116 (48) | 0.011 |
| Age, years | 69 ± 13 | 68 ± 13 | 64 ± 13 | 61 ± 13 | 76 ± 8 | <0.001 |
| Age at diagnosis, years | 51 ± 12 | 44 ± 10 | 50 ± 11 | 42 ± 8 | 60 ± 8 | <0.001 |
| Duration of diabetes, years | 18 ± 10 | 24 ± 11 | 14 ± 8 | 19 ± 10 | 16 ± 8 | <0.001 |
| Current smoker, *n* (%) | 95 (15) | 19 (15) | 23 (22) | 24 (17) | 29 (12) | 0.106 |
| Alcohol use, *n* (%) | 181 (29) | 34 (26) | 33 (32) | 39 (27) | 75 (31) | 0.704 |
| Anthropometric data | | | | | | |
| BMI, kg/m$^2$ | 26.5 ± 6.4 | 24.5 ± 4.4 | 32.3 ± 8.4 | 30.3 ± 5.3 | 22.7 ± 3.0 | <0.001 |
| Waist circumference, cm | 92 ± 15 | 89 ± 12 | 105 ± 17 | 100 ± 12 | 85 ± 10 | <0.001 |
| Systolic BP, mmHg | 132 ± 18 | 128 ± 18 | 133 ± 18 | 133 ± 18 | 131 ± 18 | 0.064 |
| Diastolic BP, mmHg | 73 ± 12 | 72 ± 12 | 75 ± 14 | 75 ± 11 | 72 ± 11 | 0.006 |
| Laboratory measurements | | | | | | |
| Fasting plasma glucose, mmol/l | 7.7 ± 2.0 | 8.9 ± 2.6 | 7.4 ± 1.7 | 7.5 ± 2.0 | 7.4 ± 1.6 | <0.001 |
| $HbA_{1c}$, mmol/mol | 54 ± 11 | 71 ± 11 | 49 ± 7 | 50 ± 7 | 49 ± 6 | <0.001 |
| $HbA_{1c}$, % | 7.1 ± 1.1 | 8.7 ± 1.0 | 6.6 ± 0.6 | 6.7 ± 0.6 | 6.7 ± 0.5 | <0.001 |
| Fasting serum C-peptide, nmol/l | 0.80 ± 0.51 | 0.55 ± 0.31 | 1.55 ± 0.63 | 0.76 ± 0.32 | 0.64 ± 0.28 | <0.001 |
| HOMA2-B | 67.7 ± 36.9 | 38.6 ± 12.8 | 128.1 ± 40.9 | 68.3 ± 18.6 | 57.0 ± 18.3 | <0.001 |
| HOMA2-IR | 1.84 ± 1.08 | 1.33 ± 0.62 | 3.71 ± 1.02 | 1.67 ± 0.57 | 1.41 ± 0.52 | <0.001 |
| Triglycerides, mmol/l | 1.2 (0.8–1.8) | 1.2 (0.9–2.0) | 1.6 (1.2–2.2) | 1.3 (0.9–2.0) | 1.0 (0.8–1.4) | <0.001 |
| HDL cholesterol, mmol/l | 1.4 ± 0.4 | 1.4 ± 0.3 | 1.3 ± 0.3 | 1.4 ± 0.3 | 1.5 ± 0.4 | <0.001 |
| LDL cholesterol, mmol/l | 2.7 ± 0.8 | 2.7 ± 0.9 | 2.7 ± 0.8 | 2.7 ± 0.7 | 2.6 ± 0.7 | 0.631 |
| AST, U/l | 21 (17–28) | 21 (17–28) | 24 (17–36) | 20 (16–28) | 21 (17–25) | 0.009 |
| ALT, U/l | 19 (14–30) | 20 (15–30) | 25 (15–41) | 20 (15–34) | 17 (12–23) | <0.001 |
| γGT, U/l | 25 (17–42) | 25 (18–43) | 38 (23–62) | 25 (17–38) | 22 (17–34) | <0.001 |
| eGFR, ml/min per 1.73 m$^2$ | 63 ± 19 | 66 ± 21 | 55 ± 21 | 68 ± 20 | 62 ± 16 | <0.001 |
| Uric acid, µmol/l | 321 ± 78 | 312 ± 82 | 359 ± 74 | 320 ± 78 | 310 ± 74 | <0.001 |
| White blood cell, $10^3$/µl | 6.3 ± 1.9 | 6.4 ± 1.9 | 6.6 ± 2.0 | 6.7 ± 1.9 | 5.8 ± 1.9 | <0.001 |
| Hemoglobin, g/l | 136 ± 18 | 139 ± 17 | 140 ± 19 | 138 ± 18 | 132 ± 17 | <0.001 |
| Platelet, $10^4$/µl | 22.2 ± 6.6 | 22.1 ± 5.6 | 21.6 ± 6.3 | 23.5 ± 6.8 | 21.7 ± 7.0 | 0.060 |
| Albuminuria, mg/gCr | 21 (8–86) | 27 (8–155) | 36 (10–180) | 21 (7–69) | 16 (7–46) | 0.003 |
| Glucose-lowering drugs, *n* (%) | | | | | | |
| Sulfonylurea | 53 (9) | 18 (14) | 5 (5) | 12 (8) | 18 (7) | 0.069 |
| Metformin | 291 (47) | 76 (59) | 44 (42) | 81 (57) | 90 (37) | <0.001 |
| DPP-4 inhibitor | 352 (57) | 73 (57) | 47 (45) | 91 (64) | 141 (58) | 0.035 |
| SGLT2 inhibitor | 157 (25) | 47 (36) | 30 (29) | 43 (30) | 37 (15) | <0.001 |
| GLP-1 receptor agonist | 64 (10) | 22 (17) | 20 (19) | 17 (12) | 5 (2) | <0.001 |
| Insulin | 184 (30) | 78 (61) | 10 (10) | 40 (28) | 56 (23) | <0.001 |

Values are presented as mean ± SD, median (IQR) or *n* (%). *p* values were obtained by one-way ANOVA, Kruskal–Wallis test or $\chi^2$ test. AST, aspartate aminotransferase; ALT, alanine aminotransferase; DPP-4, dipeptidyl peptidase-4; GLP-1, glucagon-like peptide-1; γGT, γ-glutamyl transpeptidase; SGLT, sodium–glucose cotransporter 2; T2D, type 2 diabetes

**ESM Table 3 Classification metrics for type 2 diabetes subtypes predicted using random forest algorithm**

| Dataset | Prediction model | Metrics | T2D subtypes | | | |
|---|---|---|---|---|---|---|
| | | | SIDD | SIRD | MOD | MARD |
| Test (Cohort 1) | $T2D_{RF5}$ (five Ahlqvist's fixed variables) | Accuracy | 0.940 | | | |
| | | Precision | 0.973 | 0.906 | 0.909 | 0.959 |
| | | Recall | 0.923 | 0.935 | 0.930 | 0.959 |
| | | F1 score | 0.947 | 0.921 | 0.920 | 0.959 |
| | | AUC | 0.995 | 0.984 | 0.991 | 0.990 |
| | $T2D_{RF15}$ (Boruta-selected 15 variables) | Accuracy | 0.941 | | | |
| | | Precision | 0.947 | 0.909 | 0.909 | 0.972 |
| | | Recall | 0.923 | 0.968 | 0.930 | 0.945 |
| | | F1 score | 0.935 | 0.938 | 0.920 | 0.958 |
| | | AUC | 0.994 | 0.995 | 0.991 | 0.993 |
| | $T2D_{RF25}$ (25 variables based on their routine measurement) | Accuracy | 0.930 | | | |
| | | Precision | 0.946 | 0.909 | 0.886 | 0.958 |
| | | Recall | 0.897 | 0.968 | 0.907 | 0.945 |
| | | F1 score | 0.921 | 0.938 | 0.897 | 0.952 |
| | | AUC | 0.994 | 0.995 | 0.990 | 0.994 |
| Validation (Cohort 2) | $T2D_{RF15}$ (Boruta-selected 15 variables) | Accuracy | 0.863 | | | |
| | | Precision | 0.724 | 0.922 | 0.897 | 0.948 |
| | | Recall | 0.978 | 0.922 | 0.791 | 0.829 |
| | | F1 score | 0.832 | 0.922 | 0.840 | 0.885 |
| | | AUC | 0.972 | 0.993 | 0.952 | 0.981 |
| Validation (imputed Cohort 2) | $T2D_{RF15}$ (15 variables with 3 imputed insulin-related variables) | Accuracy | 0.829 | | | |
| | | Precision | 0.737 | 0.820 | 0.831 | 0.918 |
| | | Recall | 0.963 | 0.649 | 0.786 | 0.849 |
| | | F1 score | 0.835 | 0.725 | 0.808 | 0.883 |
| | | AUC | 0.974 | 0.964 | 0.937 | 0.976 |

T2D, type 2 diabetes; $T2D_{RF5}$, T2D subtypes predicted by RF algorithm based on 5 variables; $T2D_{RF15}$, T2D subtypes predicted by RF algorithm based on 15 variables; $T2D_{RF25}$, T2D subtypes predicted by RF algorithm based on 25 variables

**ESM Fig.1 Enrollment flow chart of study participants**

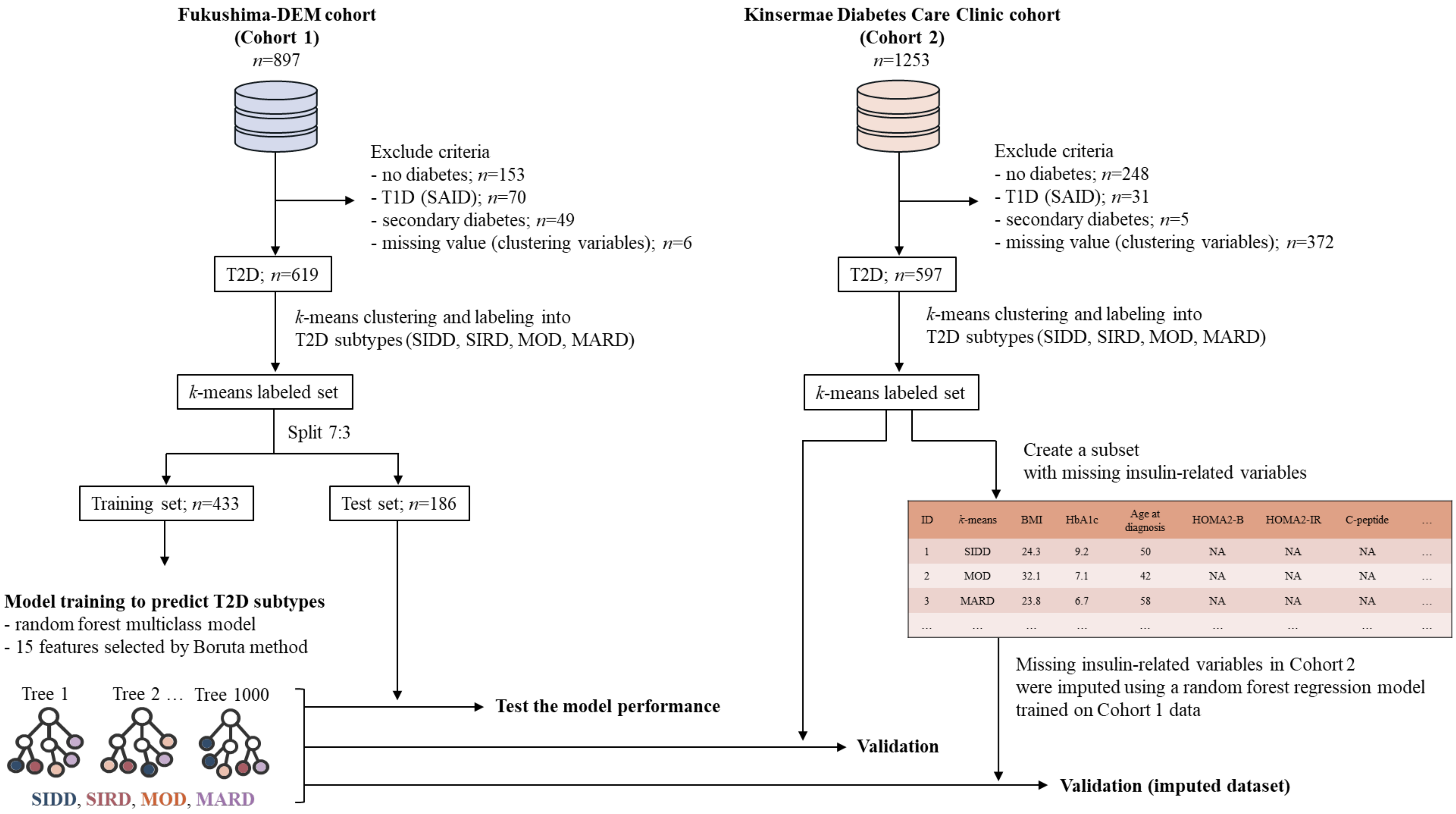


MARD: mild age-related diabetes; MOD: mild obesity-related diabetes; SIDD: severe insulin-deficient diabetes; SIRD: severe insulin-resistant diabetes; T1D, type 1 diabetes; T2D, type 2 diabetes

**ESM Fig.2 Important variables selected for each type 2 diabetes subtype using the Boruta algorithm**

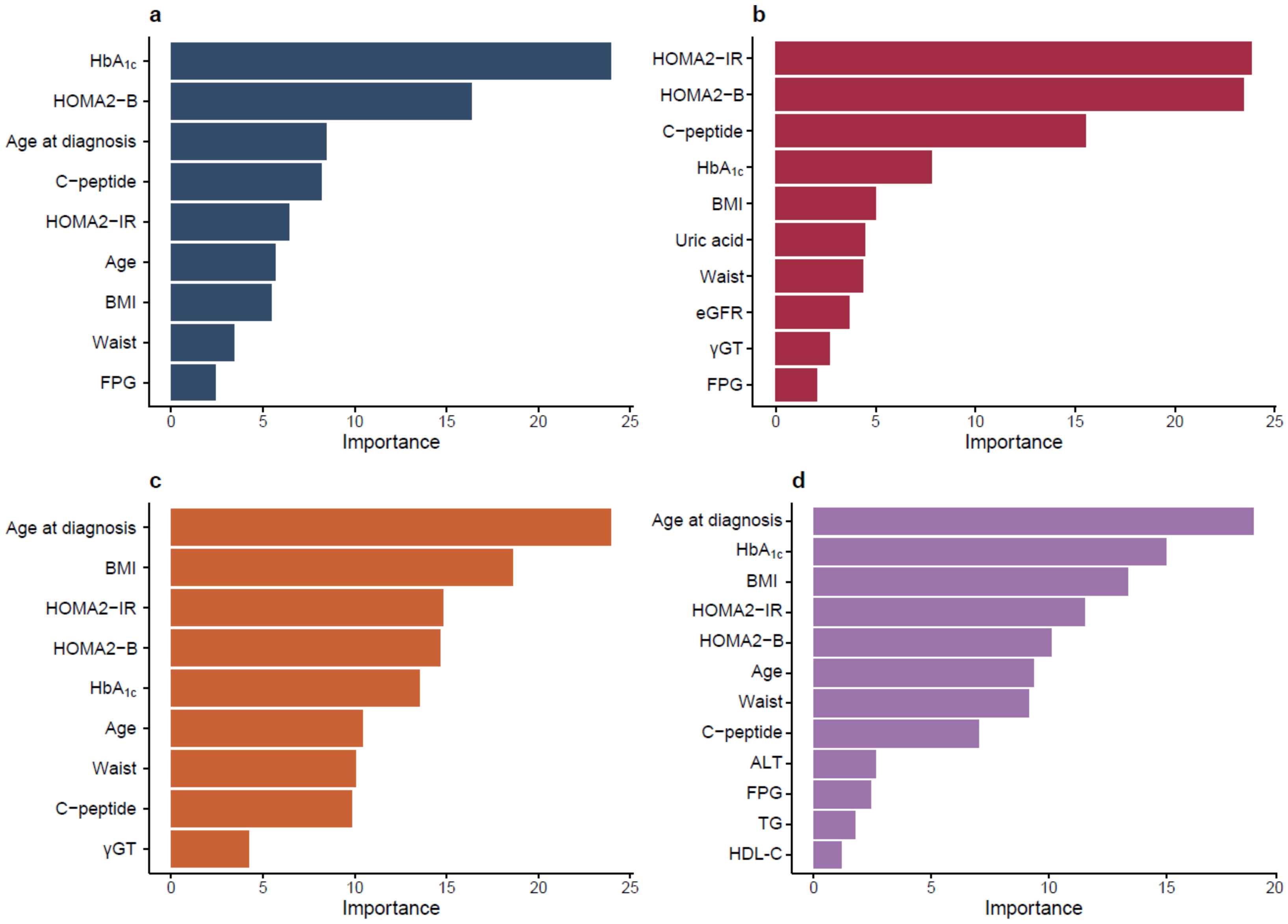


(a) severe insulin-deficient diabetes (SIDD), (b) severe insulin-resistant diabetes (SIRD), (c) mild obesity-related diabetes (MOD), and (d) mild age-related diabetes (MARD). FPG, fasting plasma glucose; ALT, alanine aminotransferase; γGT, γ-glutamyl transpeptidase

**ESM Fig.3 Profiles of predicted insulin-related variables imputed using the random forest regression algorithm in putative Cohort 2 with missing insulin-related variables**

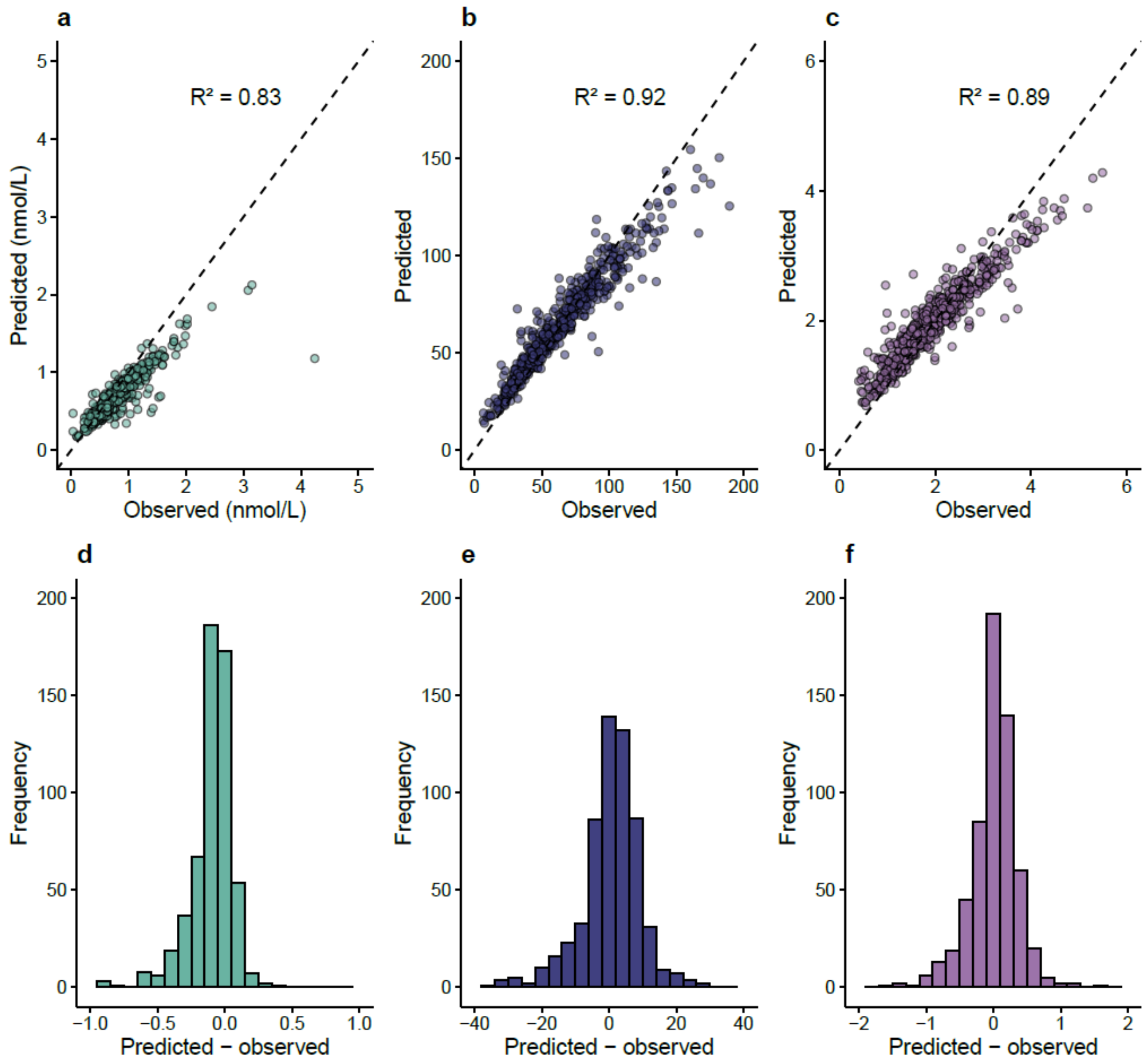


Predicted and observed values were compared, and the distribution of differences between predicted and observed values were shown in (a and d) C-peptide, (b and e) HOMA2-B, (c and f) HOMA2-IR when predictions were made in the dataset intentionally omitting those values. The dotted lines indicate y=x.

**ESM Fig.4 Impact of missing variables on classification metrics in type 2 diabetes random forest (RF) algorithm based on 15 variables ($T2D_{RF15}$)**

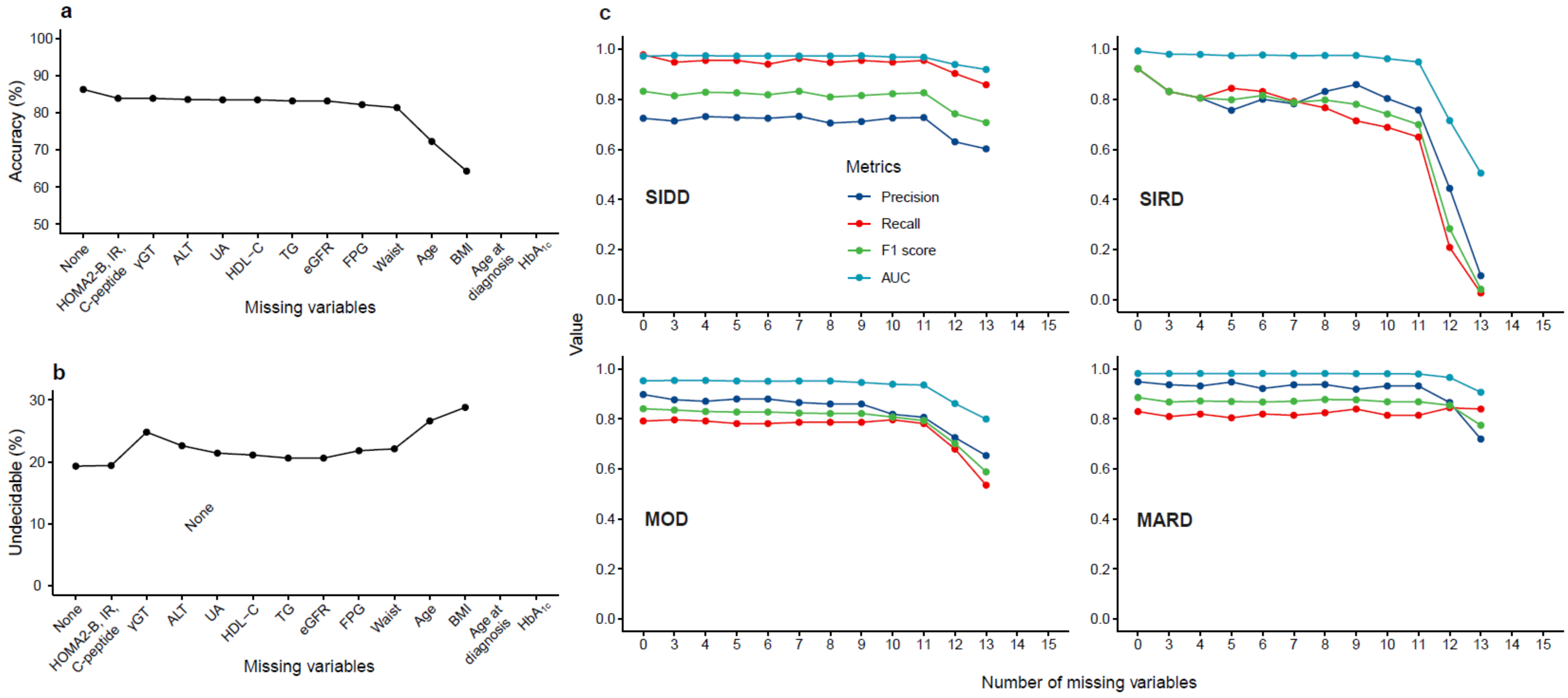


Panel a shows relationships between missing variables and prediction accuracy in overall individuals of Cohort 2. Panel b shows % proportion of undecidable individuals as a function of missing variables. Variables were omitted first for the insulin-related variables including HOMA2-B, HOMA2-IR, and C-peptide, and were thereafter sequentially based on their ascending order of importance as calculated by a RF model shown in Fig.1b. Panel c shows the classification metrics per cluster including precision, recall, F1 score, and AUC. Numbers on the x-axis correspond to those in a and b. The calculations were unavailable for age at diagnosis and $HbA_{1c}$, and thus values were not shown. ALT, alanine aminotransferase; FPG, fasting plasma glucose; γGT, γ-glutamyl transpeptidase; T2D, type 2 diabetes

**ESM Fig.5 Two dimensional (2D) visualisation of the proximity matrix with embedded individual prediction probabilities calculated by random forest (RF) algorithm based on 15 variables ($T2D_{RF15}$)**

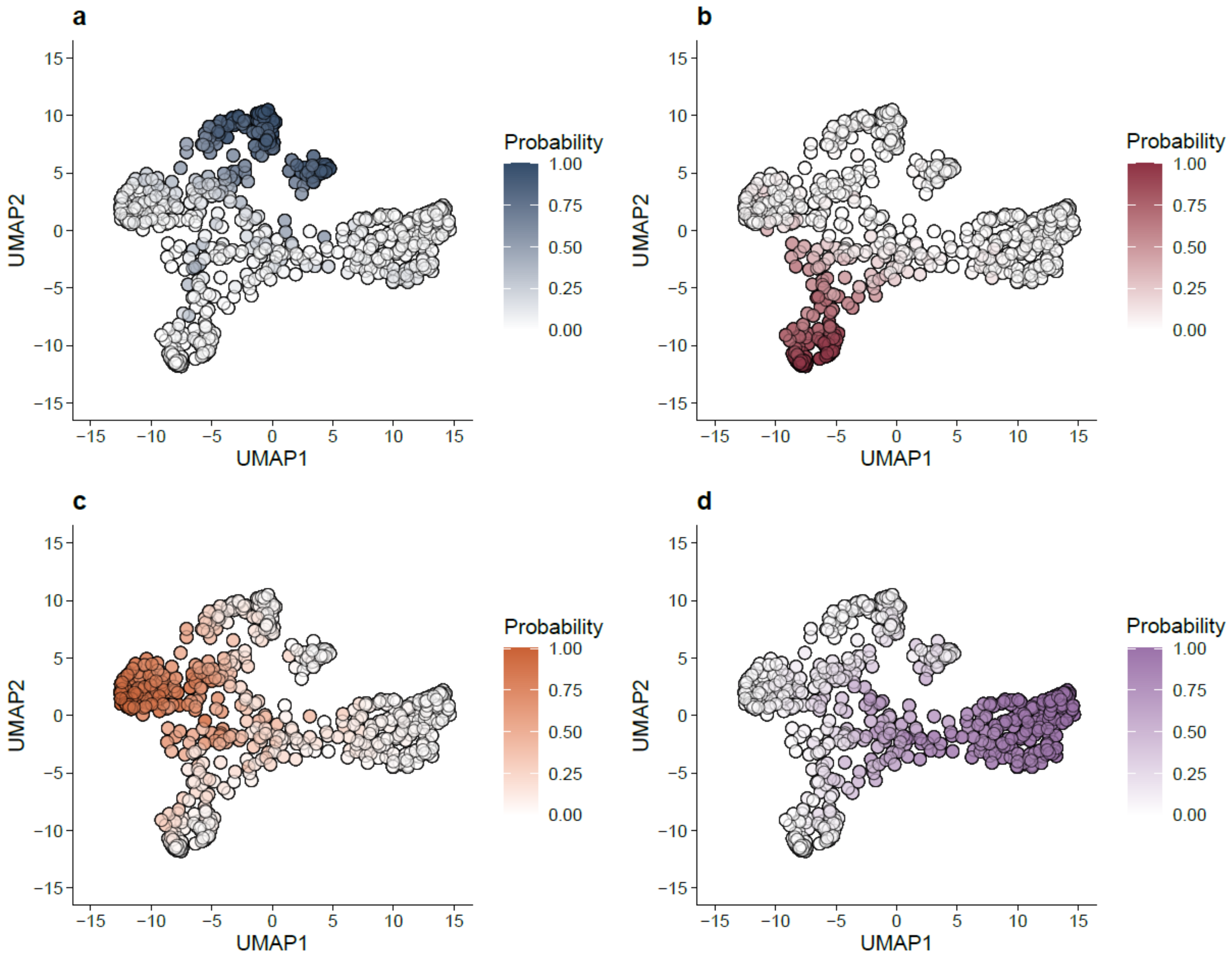


(a) severe insulin-deficient diabetes (SIDD), (b) severe insulin-resistant diabetes (SIRD), (c) mild obesity-related diabetes (MOD), and (d) mild age-related diabetes (MARD). Uniform Manifold Approximation and Projection (UMAP) was used to embed this matrix in two dimensions.

**ESM Fig.6 Migration pattern of type 2 diabetes subtype predicted by the self-normalising neural network algorithm (reported by Bello-Chavolla, et al) from baseline to 5-year follow-up for study participants in Cohort 1.**

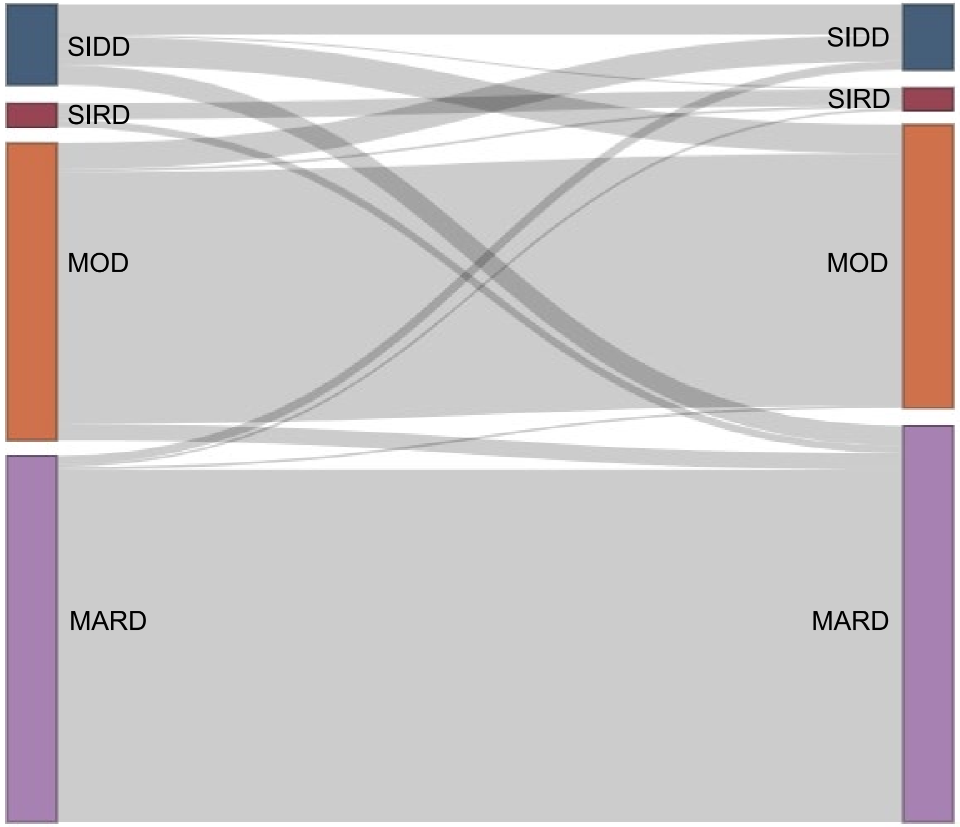


The overall performance for predicting $T2D_{kmeans}$ at baseline was 69.1%. The mean consistency over time for four type 2 diabetes subtypes was 83.8%, but the consistency was particularly low for the SIDD subtype at 37.5%.
